# Modeling Tree Growth Taking into Account Carbon Source and Sink Limitations

**DOI:** 10.3389/fpls.2017.00182

**Published:** 2017-03-21

**Authors:** Amaury Hayat, Andrew J. Hacket-Pain, Hans Pretzsch, Tim T. Rademacher, Andrew D. Friend

**Affiliations:** ^1^Department of Pure Mathematics and Mathematical Statistics, Centre for Mathematical Sciences, University of CambridgeCambridge, UK; ^2^Fitzwilliam CollegeCambridge, UK; ^3^St Catherine's CollegeOxford, UK; ^4^Technical University of MunichMunich, Germany; ^5^Department of Geography, University of CambridgeCambridge, UK

**Keywords:** tree growth, vegetation modeling, sink limitation, source limitation, height growth

## Abstract

Increasing CO_2_ concentrations are strongly controlled by the behavior of established forests, which are believed to be a major current sink of atmospheric CO_2_. There are many models which predict forest responses to environmental changes but they are almost exclusively carbon source (i.e., photosynthesis) driven. Here we present a model for an individual tree that takes into account the intrinsic limits of meristems and cellular growth rates, as well as control mechanisms within the tree that influence its diameter and height growth over time. This new framework is built on process-based understanding combined with differential equations solved by numerical method. Our aim is to construct a model framework of tree growth for replacing current formulations in Dynamic Global Vegetation Models, and so address the issue of the terrestrial carbon sink. Our approach was successfully tested for stands of beech trees in two different sites representing part of a long-term forest yield experiment in Germany. This model provides new insights into tree growth and limits to tree height, and addresses limitations of previous models with respect to sink-limited growth.

## 1. Introduction

Forests are an important component of the global carbon cycle and are currently thought to be a major sink of atmospheric CO_2_ (Bonan, 2008; Pan et al., 2011). Being able to predict the future responses of forests is therefore of great interest. Many models have been used to address this issue, but they are almost exclusively carbon source-driven, with plants at any particular location treated as a pool, or pools, of carbon mainly driven by photosynthesis (e.g., Cramer et al., 2001; Anav et al., 2013; Friend et al., 2014). However, it is likely that many other factors, such as the intrinsic limits of meristems and cellular growth rates, as well as control mechanisms within the tree, have large influences on forest responses (Körner, 2003; Fatichi et al., 2014). A few research groups have addressed the issue of sink-limited growth in a modeling context with respect to carbon sequestration. The potential for sink-limited growth to affect carbon storage and treeline position was addressed by Leuzinger et al. (2013) using a global vegetation model. However, their approach was highly empirical and only addressed temperature limitations. A more mechanistic approach was presented by Schiestl-Aalto et al. (2015), in which a tree-level carbon balance model was constructed with both carbon source and sink parameterizations. The sink parameterizations were based on thermal-time and included different ontogenetic effects between tissue types and xylogenetic processes for secondary growth. However, while this paper makes a significant contribution, the various parameterizations were very simply incorporated, with no effect of moisture on wood growth, fixed durations for xylem enlargement, and no overall tree growth across years. Gea-Izquierdo et al. (2015) also presented a tree-level model parameterization that addressed the effect of growth processes independently of photosynthesis, and in this case looked particularly at soil moisture effects. However, they did not explicitly treat meristem growth, but instead used modified allocation coefficients depending on temperature and soil water.

Grossman and DeJong (1994) examined the consequences of explicit consideration of sink-limited growth for growth partitioning through the growing season in fruit trees. Sink growth was parameterized using a priority order and as a function of carbohydrate supply up to potential rates for different tissues, which were modulated by daily temperature and season. It was concluded that source and sink limit at different times of the year, with carbohydrate supply limiting stem growth during the spring and autumn periods, presumably related to the total sink strength being maximal at those times. Derived from this type of approach, a number of so-called “Functional-Structural Plant Models” (FSPMs) have been developed which typically explicitly consider both source and sink functions (Allen et al., 2005). While the emphasis of these models has been on allocation to plant form, the focus on sinks makes them relevant for the sink-source debate, and potentially useful tools to address it. However, as far as we know they have not been directly used for this purpose. The LIGNUM model (Perttunun, 2009), for example, computes stem growth using available photosynthate in order to conform to the pipe model, based on foliage area. The tree grows as a coordinated whole, and storage is not considered. It has primarily been used to study the three-dimensional aspects of crown shape and light capture.

Most of the published growth and yield models that are routinely applied to forest ecosystem management and scenario analyses are based on statistical relationships between tree growth and environmental conditions (Pretzsch et al., 2007). Thus, they inherently consider the sink aspect of growth, in terms of parameterized resource-growth relationships, more than the source approach. Examples are the individual tree models TASS (Purves et al., 2008), SILVA (Pretzsch et al., 2002), PROGNAUS (Sterba and Monserud, 1997), SORTIE (Pacala and Deutschman, 1995), MELA (Hynynen, 2002), and HEUREKA (Wikström et al., 2011). They represent successful but maybe too exclusively sink-oriented models, in which new knowledge of tree responses to environmental conditions is difficult to integrate due to their empirical nature. In contrast, the generation of mainly source-driven models such as BALANCE (Grote and Pretzsch, 2002), TREEDYN (Bossel, 1991), and 3PG (Landsberg and Waring, 1997) represent hypotheses about biogeoeco-physiological mechanisms, but so far are little established beyond scientific applications. This is mainly due to the lack of evaluation and comparison with empirical growth and yield records. In response to this current one-sided focus on modeling either taking the sink or the source aspect into account, an integration of both seems most promising for taking forward understanding and prognosis of tree and stand growth.

Here we present a new framework for addressing tree growth responses to environmental change, building on knowledge of tree physiology to develop approaches for predicting the development of an individual tree, and thereby enabling a better understanding of forest responses to environmental change than purely source-driven models can achieve, as well as addressing the limitations of previous sink-limited approaches. Our aim is to derive an approach that can be applied in global models, specifically Dynamic Global Vegetation Models (DGVMs). Therefore, we seek the minimal level of detail necessary in order to compute behavior at the global scale, and to be compatible with the other highly aggregated process representation in DGVMs.

In this paper we suggest that this objective can be realized using differential models, that is to say growth models using differential equations. We propose here a first differential model taking into account control mechanisms and the intrinsic limits of meristems and cellular growth within trees together with the carbon balance, as well as direct environmental controls on meristematic activity. The paper is presented as follows. First we make several hypotheses concerning physiological considerations, then we give a description of the model. We show how the model is used to predict tree height and stem volume in two sets of three different stands of beech trees. Finally, we discuss the results and the behavior of the model in several situations.

## 2. Model and framework

### 2.1. General considerations

To describe the development of the tree, we suppose that it has two types of meristems, apical and lateral, and that the apical meristem increases the height through sustaining primary growth, while the lateral meristem (i.e., the vascular cambium) increases the radius through sustaining secondary growth. We recognize that trees will usually have many separate apical meristems distributed across many branches, but for convenience we treat these together as one apical meristem; we also ignore apical root growth for now. We further assume that the stem can be represented by a cylinder and that the crown (i.e., the branches and foliage) occupies a cylindrical volume and has dimensions that are proportional to the stem dimensions, i.e., the crown depth is proportional to the height of the tree stem and its radius to the radius of the tree stem. In this way we need only obtain information about the radius *r* and the height *h* of the stem to describe the growth of the tree. Future developments will include introducing other crown and stem shapes, as well as root meristems. Here our objective is to keep the model as simple as possible in order to explore the realism of its fundamental assumptions.

In order to derive the dynamics of *r* and *h* we need three types of equations:

- constitutive equations—related to the structure of the tree;
- control equations—representation(s) of the intrinsic controls that the tree has on itself;
- carbon balance equations—related to the balance of carbon between the tree and its environment, and the balance within the tree.

### 2.2. Constitutive equations: allometric relationships

Physiologically, plant growth is sustained by meristems producing new cells which subsequently enlarge and increase in mass (Aloni, 1987). We suppose that for each type of meristem, growth is proportional to its volume, that is to say that the mass of carbon allocated to a meristematic region will depend proportionally on the volume of meristem, which determines the maximum potential production rate of new cells.

We also suppose that a tree controls the activities of the meristems and therefore the relative demand for carbon *between* the meristematic regions: it can favor either height or diameter growth in this way, depending on environmental signals. Moreover, we suppose that this is the only control the tree has on its growth. With this hypothesis we soon get:

(1) $$\frac{dh}{h}=2\alpha_{2}\frac{dr}{r}$$

where α_2_ is the ratio of activities between the apical and lateral meristems, i.e., when the activity of the lateral meristem produces 1 g of carbon growth, α_2_ g of carbon growth occurs from the apical meristem. It is the control parameter.

Note that we suppose here that the volume of the apical meristem is proportional to the top surface area of the (cylindrical) stem and that the volume of the lateral meristem is proportional to the lateral surface area of the stem. This hypothesis is used as it is more realistic than assuming that the volume of the lateral and apical meristems are proportional to the volume of the tree, i.e., that the thickness of the lateral meristem area increases proportionally with the radius and the thickness of apical meristem increases proportionally with height. Although even under this alternative hypothesis we would get the same type of equation with α_2_ being the proportion of growth per unit of volume of apical meristem relative to proportional lateral growth, only adding a numerical coefficient in front of α_2_.

Knowing the definition of the volume and integrating we get the following constitutive equations:

(2) $$h=\gamma r^{2\alpha_{2}}$$

(3) $$M_{\text{c}}=g_{1}r^{2}h$$

We recognize Equation (2) as the usual allometric relationship where γ is a constant. As it is true for any time *t*, it can be expressed as $h_{0}/r_{0}^{2\alpha_{2}}$, with *h*_0_ and *r*_0_ the height and radius at some time origin *t* = 0.

Equation (3) is simply the definition of the volume of the stem plus canopy (containing the branches and leaves) in relation to its mass, *M*_c_. We assume naturally that branches, stem, and leaves have different densities, but for each of them we suppose that their mass is proportional to *r*^2^ × *h* with potentially a geometrical coefficient. That implies that the general mass is proportional to *r*^2^ × *h*, and in Equation (3), *g*_1_ is the global proportionality coefficient. Therefore, *g*_1_ takes into account the density of the stem, the density of the branches and foliage, their contribution to the mass of the tree, and geometric factors. *g*_1_ can therefore be found using the proportionality coefficients we assumed between crown radius and stem radius and crown height and stem height, and their relative densities. Or if we call *a* the geometrical factors and *d* the densities: *g*_1_ = *a_foliage_d_foliage_* + *a_branches_d_branches_* + *a_stem_d_stem_*. Here *a* takes into account the geometry and the volume of each element.

### 2.3. Control mechanism of the tree

We want now to take into account the intrinsic control that the tree has on itself, parameterized in our model by α_2_, the ratio between the activities of the apical and lateral meristems. For this we need to consider what happens physiologically (for convenience we use teleological terminology): the tree uses internal controls to be able to adapt its shape and physiology to the surrounding environment, for instance other competing trees (e.g., Ritchie, 1997), through phytohormonal signaling networks (Brackmann and Greb, 2014; Aloni, 2015). Many behaviors could be taken into account, but here we focus on the behavior of a tree competing for light by trying to grow taller faster than its competitors by increasing α_2_. The modeled tree is assumed to detect the presence of surrounding trees by sensing the ratio of downwelling red radiation (i.e., wavelengths between 655 and 665 nm) to downwelling far-red radiation (i.e., wavelengths between 725 and 735 nm). A low ratio signals potentially more neighboring trees and therefore a potential threat of shading. In that case the tree reacts by increasing the activity of the apical meristem relative to the lateral meristem, while a higher ratio potentially means no threat and so the tree reacts by balancing the activities of the two meristems as it needs to compete less for light (Franklin, 2008). We hypothesize that this reaction can be modeled as:

(4) $$\alpha_{2}=\alpha_{0}\exp(-\alpha_{3}R_{\text{a}})$$

Where α_0_ is the highest limit for α_2_ beyond which the tree breaks due to mechanical failure, and *R*_a_ is the ratio of received downwelling red:far-red ratio. α_3_ is a scaling parameter such that α_0_ exp(−α_3_) is the value of α_2_ when the tree is unshaded (i.e., when it detects no other tree around).

This approach is based on the findings of several studies (e.g., Morgan and Smith, 1976; Franklin, 2008). In particular, Morgan and Smith (1976) observed the laboratory behavior of two similar young trees grown for 21 days with the same intensity of photosynthetically active radiation (PAR), but different red:far-red ratios. They experimentally obtained a relationship which, extended by limited development for short trees, is coherent with the relationship we give in Equation (4).

A potential limitation to our approach is that we may not have access to direct measurements of *R*_a_. For a lone tree this would not be a real problem: the allometric relationship would be constant in our model and the red:far-red ratio maximal. For individual trees growing in a stand in a forest, however, the situation is different as the trees shade each other.

Finding a dependency rule of the red:far-red ratio for trees in a stand is therefore a challenge that does not seem to be overcome yet. Because of this and our desire to keep the model simple^1^, when we do not have access to measurements of the red:far-red ratio we make the assumption that α_2_ is constant for each stand, as it would be for a lone tree. When the value of this constant is required, it will be estimated as a typical value consistent with the empirical allometric relationship. This is likely to be our most limiting factor as we are here trying to model the internal control that the tree has on itself, which is probably the most complicated phenomenon to take into account, and likely the least-well understood component of our model (cf. Li et al., 2011; Aloni, 2015). Although the model seems to work realistically, further work may require a more precise dependency of the red:far red ratio.

### 2.4. Carbon balance

The variation of carbon in the tree over time can be written as the sum of the carbon used for volume growth and the carbon stored:

(5) $$\frac{dM_{\text{tot}}}{dt}=\frac{dM_{c}}{dt}+\frac{d((S-S_{1})\frac{g_{1}}{\left(1+S_{1}\right)}\pi hr^{2})}{dt}$$

where *M*_*c*_ is the carbon used for volume growth, $S_{c}=S\frac{g_{1}}{\left(1+S_{1}\right)}\pi hr^{2}$ the carbon stored, *S* is defined as carbon stored per unit volume of stem, $S_{c_{0}}=\left(S-S_{1}\right)\frac{g_{1}}{\left(1+S_{1}\right)}\pi hr^{2}$ is the carbon stored above the storage limit *S*_1_ under which the tree is considered in danger. This distinction has a physical interpretation and therefore in this model the density of the tree can then be seen as a standard density related to the carbon used for growth plus an increase of density due to the storage.

We also know that the variation of carbon is given by:

(6) $$\frac{dM_{\text{tot}}}{dt}=A(Q1,C_{g},T,\psi ,h,r)-C_{1}hr^{2}-\frac{C_{2}(1+S_{1})}{g_{1}\pi }S_{\text{c}}$$

where *A* is the carbon assimilation due to gross photosynthesis minus a foliage respiration part proportional to photosynthesis, while *C*_1_ is a constant grouping a part of the respiration and a litter component proportional to the volume, together with the geometrical factor *g*_1_π (such that *C*_1_/*g*_1_π is the respiration and litter component constant). We assume here that there is no other litter component, that is to say that litter is completely proportional to the volume of the stem. We note that this also means that the litter is proportional to the volume of the crown with our previous assumptions. Also we assumed here that we are dealing with trees where the living volume can be approximated as proportional to the total volume. This is an approximation and further work could include a variable corrective coefficient, however this is a secondary issue compared to the main advance of this model concerning sink-limited growth. Finally, *C*_2_ is the same type of constant but for the carbon storage. *A* is assumed to depend on PAR (*Q*1), the concentration of CO_2_ in the atmosphere (*C*_*g*_), temperature (*T*), water potential (ψ), and the dimensions of the tree (*h* and *r*).

Therefore:

(7) $$\frac{dM_{c}}{dt}+\frac{dS_{{\text{c}}_{0}}}{dt}=A(Q1,Cg,T,\psi ,h,r)-Chr^{2}-\frac{C_{2}(1+S_{1})}{g_{1}\pi }S_{\text{c}}$$

The system is so far incomplete as there needs to be a relationship that controls which part of the carbon is allocated to growth and which part is allocated to storage. Also, the volume growth of the tree is sustained by meristematic cell division and enlargement, with a maximal rate determined by intrinsic physiological limits or environmental controls.

We assume that the tree grows in volume as much as it can, as long as it keeps enough storage to avoid being in danger, such as for repairing damage and surviving poor growing seasons. We assume that there is one tree specific storage pool, although we acknowledge that localized storage with various turnover times are more realistic (Sprugel et al., 1991; Palacio et al., 2014; Richardson et al., 2015). Therefore, if there is enough carbon assimilation then the rate of growth is equal to the meristem-sustained growth-rate limit and the storage can increase using the difference of these two. Also, if the carbon assimilation rate becomes too low but there is enough storage, then the storage delivers carbon to the meristematic region to maintain a rate of growth equal to its maximal sustainable rate. Then density decreases as the carbon of the storage is used to maintain the volume growth. This is likely to occur for instance when buds appear while photosynthesis is not yet high enough to support the maximal growth potential due to meristem reactivation (note that bud-burst and other seasonal phenological phenomena are not yet treated by the model). However, if there is not enough carbon assimilation or storage then the rate of growth is equal to the carbon assimilation rate and the storage per unit volume remains constant.

This behavior can be translated into evolution equations. As growth occurs at a cellular level we can assume that the maximal meristem growth is proportional to the volume of meristems. We denote this maximal meristem-sustained growth per volume by *R*_max_, the volume of the meristems by *V*_me_.

If there is enough photosynthesis or storage, i.e.,

(8) $$\text{if }A(Q1,Cg,T,\psi ,h,r)-Chr^{2}-C_{2}Shr^{2}>R_{\text{max}}V_{\text{me}}\text{ or }S>S_{1}$$

then:

(9) $$\frac{dM_{c}}{dt}=\frac{R_{\text{max}}V_{\text{me}}}{1+\frac{\left(S-S_{1}\right)}{\left(1+S_{1}\right)}}$$

(10) $$\begin{array}{l} \frac{d((S-S_{1})\pi \frac{g_{1}}{\left(1+S_{1}\right)}hr^{2})}{dt}=A(Q1,Cg,T,\psi ,h,r)-Chr^{2}\\ \;-C_{2}Shr^{2}-\frac{dM_{c}}{dt} \end{array}$$

(11) $$\begin{array}{l} \text{but if }A(Q1,Cg,T,\psi ,h,r)\;-\;Chr^{2}-\;C_{2}Shr^{2}\leq R_{\text{max}}V_{\text{me}}\\ \text{ and }S\;=\;S_{1} \end{array}$$

then

(12) $$\frac{dM_{c}}{dt}=A(Q1,Cg,T,\psi ,h,r)-Chr^{2}-C_{2}Shr^{2}$$

(13) $$\frac{dS}{dt}=0$$

Note that the case where for some reason the carbon assimilation by photosynthesis is insufficient even to cover the cost of maintenance is treated by Equations (8–10). In that case there will be a depletion of storage to sustain the maintenance cost until the storage reaches a critical level or until the photosynthesis rate increases sufficiently.

### 2.5. Modeling photosynthesis

We assume the following dependencies of photosynthesis on water potential, atmospheric CO_2_ concentration (*Cg*), PAR (*Q*_1_), temperature, radius, and height:

1. Photosynthesis increases with CO_2_ concentration and PAR exponentially and tends to saturate:
  (14) $$A\;\alpha \;(1-\exp(-\frac{Cg}{Cr}))$$
  (15) $$A\;\alpha \;(1-\exp(-\frac{Q_{1}}{Q_{r}}))$$
  *Cr* and *Q*_*r*_ refers to the critical constants.
2. As a first approximation photosynthesis of each leaf decreases linearly with a reduction in soil water potential. We also suppose that water potential and hence photosynthesis decreases linearly with height (Friend, 1993):
  (16) $$A_{\text{leaf}}\;\alpha \;\frac{h_{1}-h}{h_{1}}$$
  Where:
  (17) $$h_{1}=\frac{\left(\psi_{\text{soil}}-\psi_{\text{lim}}\right)}{R_{\text{h}}}$$
  With ψ_soil_ the water potential in the soil and ψ_*lim*_ the limit water potential below which any photosynthesis cannot be performed due to a lack of turgor pressure in the leaves and increased probability of xylem cavitation. *R*_*h*_ is the hydraulic resistance per unit length. Overall for the whole canopy we suppose a dependency of the form:
  (18) $$A\;\alpha \;(\frac{h_{1}-\beta h}{h_{1}})h$$
  In particular this means that there are two antagonist effects: one that tend to increase photosynthesis with the depth of the canopy due to more leaves and another that tends to make photosynthesis decrease with height due to the decline in water potential. Note that β is a numerical coefficient that depends on the ratio between the crown depth and the height. We assumed in our simulations that β = 3/4. Also, the dependency of both height and water potential is given above.
3. We assume a dependency with temperature given by an asymmetric parabola:
  (19) $$A\;\alpha \;(1-(\frac{\left|T-T_{\text{opt}}\right|}{T_{\text{i}}}{)}^{2})$$
  With *T*_i_ being 21°C if *T* > *T*_opt_ and 25°C otherwise. We set here *T*_opt_ = 18°C. These values are inspired by previous studies (e.g., Precht et al., 1973).
4. We suppose that photosynthesis is proportional to the surface area of the crown, and so proportional to *r*^2^.

Overall we get:

(20) $$\begin{array}{l} A=A_{\text{max}}(1-e^{-\frac{Cg}{Cr}})(1-e^{-\frac{Q_{1}}{Qr}})(1-(\frac{\left|T-T_{\text{opt}}\right|}{T_{\text{i}}}{)}^{2})\\ \;(\frac{h_{1}-3h/4}{h_{1}})r^{2}h \end{array}$$

### 2.6. Maximal meristem-sustained growth

Intuitively, as meristem-sustained growth occurs at a cellular level, we assume that the limit to meristem-sustained growth per volume increases with temperature up to a certain temperature and increases with water potential (cf. Deleuze and Houllier, 1998). Therefore, we assume the following dependencies:

1. Themeristem-sustained growth limit decreases with height as: $R_{\text{max}}\;\alpha \;\frac{\left(h_{2}-h\right)}{h_{2}}$
  (21) $$h_{2}=\frac{\left(\psi_{\text{soil}}-\psi_{\text{lim}2}\right)}{R_{\text{h}}}$$
  With ψ_soil_ the water potential in the soil and ψ_lim2_ the limit water potential below which meristem-sustained growth cannot occur. Physically this gives us a maximal height *h*_2_ due to the limit on meristematic growth.
  We define therefore *R*_max0_ the reference maximal meristem-sustained growth per volume independant of water potential by: $R_{max}=R_{max0}\frac{\left(h_{2}-h\right)}{h_{2}}$
  We could also suppose that the limit to meristem-sustained growth per volume increases with temperature using a standard *Q*_10_ formulation: $R_{max}\;\alpha \;Q_{10}^{\frac{T-T_{2}}{10}}$. However, seen the difficulty to know precisely *Q*_10_ and in order to keep the model as simple as possible we assumed that *R*_max0_ remains constant with time.
2. As stated previously, we suppose that the volume of meristems is expressed as: $V_{me}=\left(t_{1}\pi r^{2}+2\pi rht_{2}\right)$, where *t*_1_ is the effective thickness of the apical meristem and *t*_2_ is the effective thickness of the lateral meristem.

### 2.7. Final evolution equations

Using the previous elements we finally get:

(22) $$\frac{dM_{c}}{dt}=\frac{R_{\text{max}0}(\frac{h_{2}-h}{h_{2}})V_{\text{me}}}{1+\frac{\left(S-S_{1}\right)}{\left(1+S_{1}\right)}}$$

(23) $$\begin{array}{l} \frac{dS_{{\text{c}}_{0}}}{dt}=(A_{\text{max}}(1-e^{-\frac{Cg}{Cr}})(1-e^{-\frac{Q1}{Qr}})(1-{\left(\frac{\left|T-T_{\text{op}}\right|}{T_{\text{i}}}\right)}^{2})\\ \;(\frac{h_{1}-3h/4}{h_{1}})-C)hr^{2}-C_{2}Shr^{2}-\frac{dM_{c}}{dt} \end{array}$$

or

(24) $$\begin{array}{l} \frac{dM_{c}}{dt}=(A_{\text{max}}(1-e^{-\frac{Cg}{Cr}})(1-e^{-\frac{Q_{1}}{Qr}})(1-{\left(\frac{\left|T-T_{\text{op}}\right|}{T_{\text{i}}}\right)}^{2})\\ \;(\frac{h_{1}-3h/4}{h_{1}})-C)r^{2}h-C_{2}Shr^{2} \end{array}$$

(25) $$\frac{dS}{dt}=0$$

depending on whether carbon supply from photosynthesis plus storage is sufficient to meet demand for growth (Equations 22 and 23) or not (Equations 24 and 25).

### 2.8. Deriving the values of the parameters

So far we need to know the values of the following physiological parameters to use the model (Table 1):

$$A_{\text{max}},\;g_{1},C,\;C_{1},\;C_{2},\;S_{1},\;Cr,\;Qr,\;h_{1},\;h_{2},\;t_{2}R_{{\text{max}}_{0}}$$

**Table 1:** **Numeric values of the parameters used in the model**.

**Symbol**	**Value**	**Quantity**
*g*_1_	365 *kg*.*m*^−3^	Density of carbon of the equivalent cylinder representing the tree
*C*/π	73 *kg*.*m*^−3^.*y*^−1^	Respiration and litter coefficient
*C*_2_/π	73 *kg*.*m*^−3^.*y*^−1^	Respiration coefficient for the storage
*t*_2_*R*_max0_/*g*_1_	0.0201 *m*.*y*^−1^ corresponds to a maximal radius expansion of 1.5 *cm*.*y*^−1^	Reference maximal meristem-sustained lateral growth per area independent of water potential
*t*_2_/*t*_1_	1	Ratio between lateral and apical meristem thickness
β*h*_1_	67.5 *m*	Limit height of occurrence for photosynthesis
*h*_2_	47 *m*	Limit height of occurrence for meristem-sustained growth
*S*_1_	0.2	Critical value of the storage per unit mass
*A*_max_/π	206 *kg*.*m*^−3^.*y*^−1^	Maximal assimilation of carbon per unit volume by photosynthesis
*Q*_*r*_	1000 *W*.*m*^−2^	Characteristic PAR dependency
α_2_	0.34–0.43	Ratio of meristematic activity
*C*_*r*_	500 ppm	Characteristic CO_2_ mixing ratio dependency
β	3/4	Geometrical integrand for *h*_1_
*T*_opt_	18°C	Optimal temperature for carbon assimilation

The most accessible are probably *Cr* and *Qr*. These are usually well known and can be obtained from the curve of photosynthesis activity as a function of atmospheric CO_2_ mixing ratio (in ppmv) or as a function of PAR, respectively.

*A*_max_ can be obtained by direct measurements of the carbon exchange at a given height, ambient CO_2_ concentration, temperature, PAR, and compensating for respiration as in Campioli et al. (2011). Acting similarly and measuring litter with litter traps gives access to *C*. These values can also be deduced from net primary production and gross primary production and the typical litter flux rates. Note that here we use data coming from forest stands, although in the model we suppose that we have an individual tree without shading because data for lone standing trees are seldom available. This means that we may underestimate the growth of isolated trees, but at the same time the values of *A*_max_ and *C* obtained enable to take into account the shading effect by neighbors of a forest with the same density, which is a hidden parameter of this model (i.e., not explicitly taken into account). Also, depending on the typical time length of the study, we choose a model timestep and therefore we use averaged values of the parameters on this timestep. In this analysis, we chose 1 year as the timestep although smaller timesteps could be considered.

Finding *h*_1_ is equivalent to finding the minimal water potential that can sustain photosynthesis. *h*_2_ is its equivalent with respect to growth. Combined, these two parameters take into account all water potential-dependant limits in trees such as cavitation in the xylem conduits, minimal turgor potential to maintain the shape of the leaf and enable stomatal opening, etc. While *h*_1_ (or equivalently ψ_max1_) has been extensively studied (e.g., Friend, 1993; Koch et al., 2004; Du et al., 2008), and is believed to be equal to around 90 m for redwood trees (Koch et al., 2004), *h*_2_ seems to be less known. However, knowing *h*_1_ and the other parameters, as well as the effective limit height for the considered species, *h*_2_ can be estimated by assuming that it is the limiting parameter in regions with very good conditions for photosynthesis. Therefore, looking at the effective limit height would give access to *h*_2_ for these particular regions. Using Equation (22) and regressing on the water potential we would have access to ψ_*lim*_ and *R*_*h*_ and therefore we would be able to estimate *h*_2_ in any region from a measure of ψ_*soil*_.

We can find *R*_*max*0_, at least approximately, by looking at the tree rings of many trees across several geographical locations and assuming that the largest ones correspond to the maximal increment *dr* achievable in a year, which is directly linked to *R*_max0_, the maximal meristem-sustained growth rate under perfect water potentials. Then we can deduce the value of *R*_max0_, or at least its order of magnitude.

## 3. Results

The model described above is ordinary, first order, non-linear, and has no widely known explicit analytical solution in the case where 2α_2_ is not an integer. Therefore, it was solved numerically by the Runge-Kutta-Fehlberg (RK45) method (Dormand and Prince, 1980). Coherence both with the analytical solution in simplified cases and with other solving methods (i.e., the trapezoidal rule and numerical differentiation formula) was tested.

As some parameters can depend, *a priori*, on the considered species, we derived the parameters explained previously for beech trees from the data provided in Campioli et al. (2011) and Zianis and Mencuccini (2005). Beech trees were chosen because of their importance in European forests, especially in Germany where they represent more than 15% of the forest and in France where they comprise 15% of the non-conifer forest. Therefore, a great number of studies have focussed on beech trees, enabling easier comparisons with our current study.

As the computation of the parameters is based on independent measurements that have sometimes a non-negligible margin of error, we allowed the parameters thus obtained to vary with a 20% margin to account for this error, and to allow a small adaptation of the model (as it is only a simplification of reality) by fitting them with reference measurements of a standard beech tree stand in Fabrikschleichach, Germany that we refer to as “first stand” in the following. As *R*_max0_ was more coarsely estimated we allowed it a variation of 50% in order to maintain the right order of magnitude. Also, as no precise data on the soil water potentials over the tested period A.D. 1870–2010 were available for these stands, we allowed a 50% variation of *h*_2_, the height where meristem-sustained growth cannot occur due to cell turgor limits. We obtained then a final vector of parameters for our model using a non-linear least-square method (trust-region-reflective algorithm: http://uk.mathworks.com/help/optim/ug/equation-solving-algorithms.html).

We then used our model to predict the time variation of height and volume of an average tree in other stands of beech trees. It should be emphasized that there is a substantial difference between fitting and prediction. Fitting a model to some data gives a partial understanding of the data but does not usually enable prediction for another tree or even for future data points as the fitting is done on a restricted set of measurements. Prediction, on the other hand, is much harder to achieve as it supposes the use of previously-derived parameters and environmental information (e.g., air temperature, soil moisture, CO_2_ concentration, etc.) to predict the measurements. Usually most models are fitted rather than predicting as it is much harder to predict anything without any fitting, although when it works it gives much more information: a better understanding and a reliable way to deduce future measurements before they occur even for other trees (e.g., height or volume prediction).

On these stands the model was tested without any additional fitting. In that sense it is, at least partially, a prediction. Of course a fitting on these stands would give a better result than the (still good) results we obtain, however it is not the goal of our approach. This is the reason why in the figures, to emphasize the difference of treatment between the standard stand and the others, two labels appear: “partial fit” and “prediction.”

**Figure 1:**
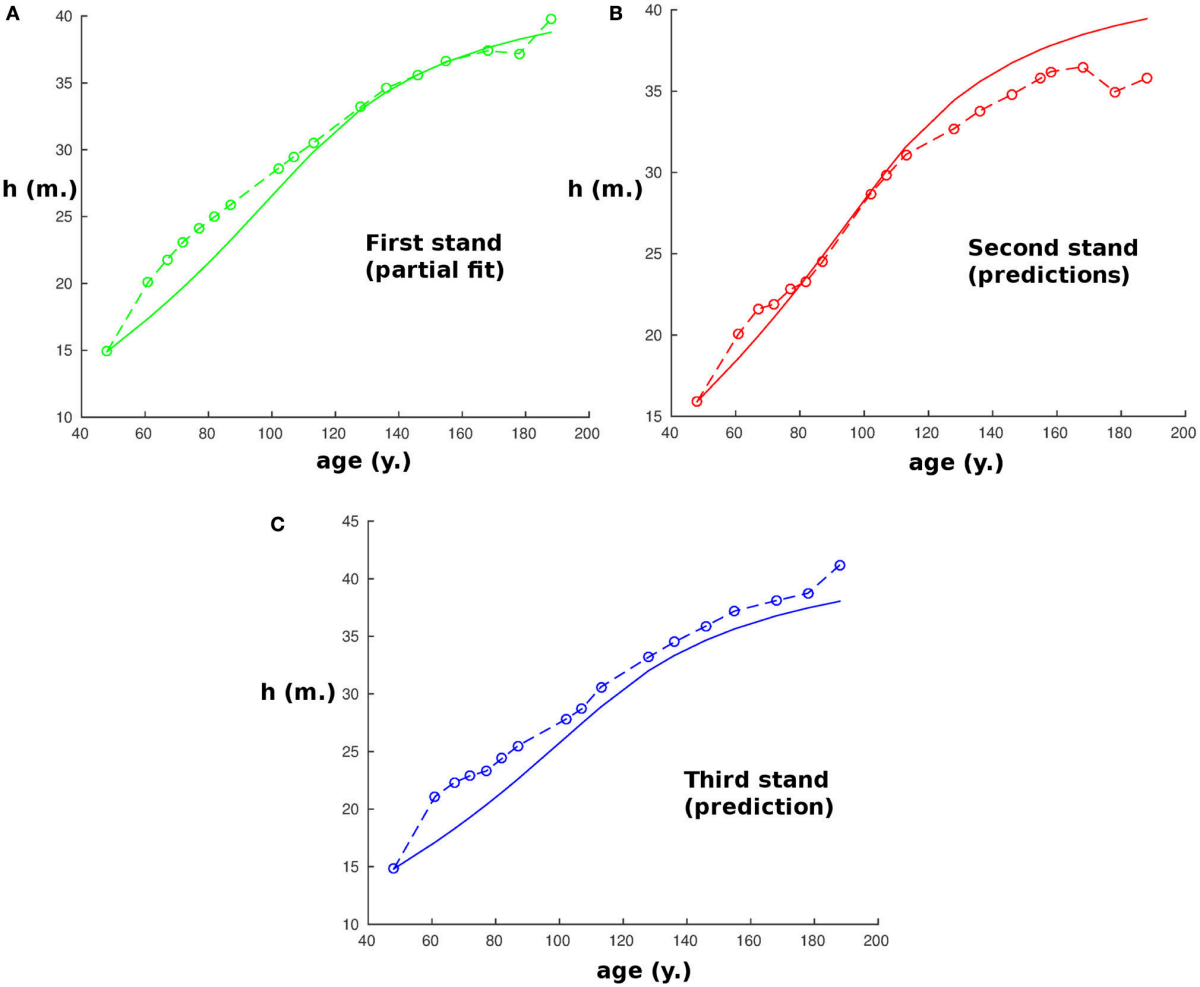
**Size of the modeled tree with age at the first site (Fabrikschleichach)**. Continuous curves are computed with the model while circles correspond to experimental measurements. **(A)** Represents the results of the partial fit on the reference stand. **(B,C)** Represent the results of the prediction with the parameters obtained by partial fitting.

We first considered three stands of beech trees that are part of a long-term experiment on forest yield science performed by the Chair of Forest Growth and Yield Science at the Technische Universität München. The stands are in southern Germany, about 60 km from Würzburg in the heart of the Steigerwald, a richly forested hillside, near a small village named Fabrikschleichach (49.9 °N; 10.5 °E). They cover roughly 0.37 ha, are located within immediate proximity of each other (i.e., they have experienced the same environmental conditions), and differ only in silvicultural management, especially thinning with consequences for the development of stem densities during the experiment. The beech trees were planted and then measured at irregular intervals averaging 8 ± 3 (mean ± standard deviation) years from an age of 48 y in A.D. 1870 to 188 y in A.D. 2010. For each stand we considered the average height of the 100 trees with the largest stem diameter at breast height. The first stand was the reference stand used to partially fit the parameters (within the 20% margin). As the model needs initial values for both *h*_0_ and *r*_0_, and as measurements only started at an age of 48 y, all the computations were run starting at an age of 48 y as can be seen in Figure 1.

As expected, our partially fitted model agrees well with the measurements from this stand (Figure 1A; *R*^2^ = 0.924). Predictions with the model were then performed for the two other stands (Figures 1B,C).

The results give a very good prediction for the second stand (*R*^2^ > 0.925), and a small, although non-negligible, error for the third one (*R*^2^ = 0.89). However, this could be at least partially explained by the change in the density of trees during the 120 y due to different rates of thinning in the different stands (i.e., from 3,500 tree *ha*^−1^ to 200 tree *ha*^−1^ in the first stand, 6,400 tree *ha*^−1^ to 200 tree *ha*^−1^ in the second, and 2,400 tree *ha*^−1^ to 200 tree *ha*^−1^ in the third). Therefore, the red:far-red radiation ratio changes with time in the real forest while it was assumed constant in our model.

**Figure 2:**
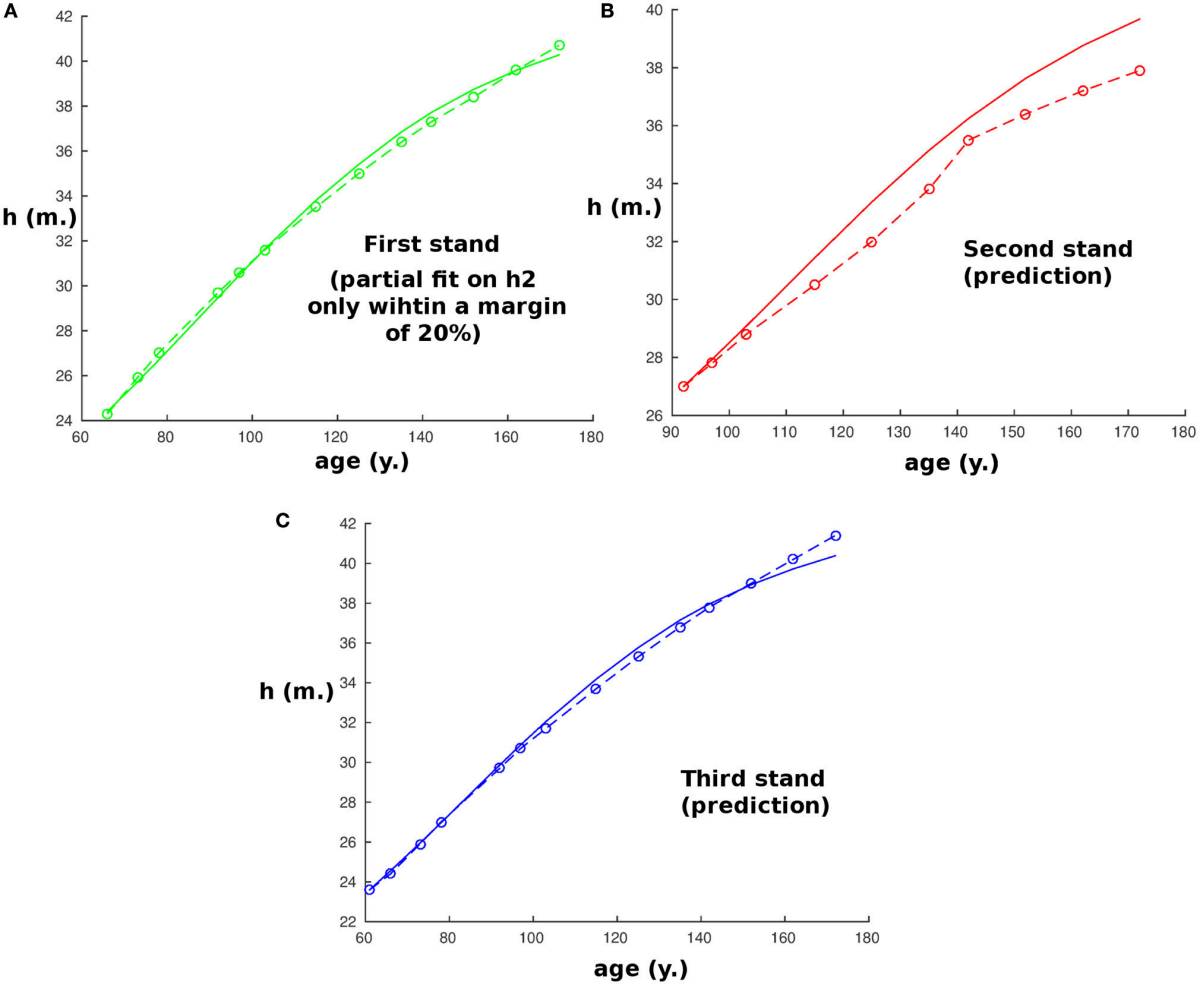
**Plot of the size of the tree with age at the second site (Hain)**. Continuous curves are computed with the model and start when the tree density is lower than 1,000 tree ha^−1^, while circles correspond to experimental measurements. Parameters for beech trees are obtained from the first site and *h*_2_ was allowed to vary up to 20% to compensate for the lack of data concerning water potential from A.D. 1870 on the two sites. Panels **(A–C)** represent, respectively, the results for stands 1–3.

To complete the analysis by testing the model thus calibrated on another site where the conditions may be different, it was also used to predict the height of dominant trees in three other stands at a different site in the Spessart, Hain (50.0 °N; 9.3 °E), a low wooded mountain range in central Germany, located approximately 100 km from the first site where the beech trees were also planted and then measured from an age of 48 y in A.D. 1870 to 188 y in A.D. 2010 (Figure 2). As data were missing concerning the water potential during the growth period from A.D. 1870 to compare with the first stand, we allowed the model to adapt its value of *h*_2_ within 20% by keeping the calibration derived previously and fitting only this one parameter on the first of the three new stands. Then the new calibration was used to predict the heights of the three stands according to the model. To avoid the potential error induced by the different variations of the red:far-red ratios within the stands, we restrict our analysis to data when the density was lower than 1,000 tree *ha*^−1^ and started the simulations from these ages. As before, the predictions are in strong accordance with the observations for all three stands, with a nearly exact correspondence with the measurements (*R*^2^ = 0.99, *R*^2^ = 0.92, *R*^2^ = 0.99 respectively), except for the second stand.

We should note that the correspondence is very close but not exact, and that the data we have might limit the accuracy: firstly, from our equations we can see that there is a propagation of error from the initial conditions. An error on the initial height of 1 m could imply an error of the predicted height of up to 2.5 m after 50 years even if the model were perfect. Also, no data were available for the difference of water potentials between the stands on a same site, while it seems logical that for a certain density of trees, the stands with higher density will have less water available per tree. Therefore, this could induce a small error in *h*_2_ and *h*_1_ that could be corrected if we knew the average water potential during the growth period in each stand.

**Figure 3:**
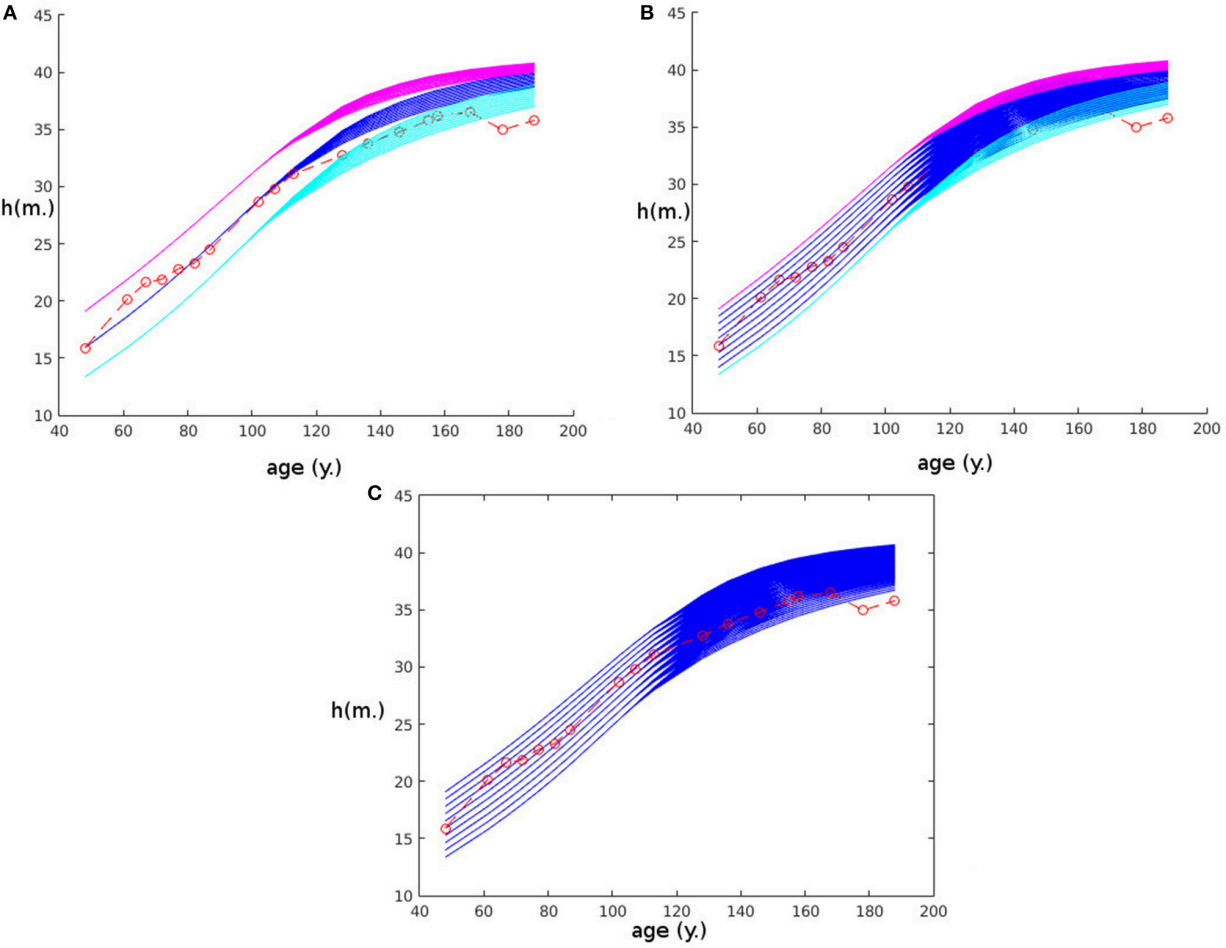
**Plot of the size of the tree with age at the second stand of the first site (Fabrikschleichach) when some error is added to the initial conditions**. On every plot the error on *r*_0_ ranges between −20 and +20%, while the error on *h*_0_ depends on the plot. **(A)** Three groups of initial conditions are represented: no error on *h*_0_ (blue), -20% error on *h*_0_ (magenta), +20% error on *h*_0_ (cyan). **(B)** Error on *h*_0_ ranges between −20% (cyan) and +20% (magenta) with a 4% increment. **(C)** Same as the previous but, in addition, allometric relationship of the stand is assumed unknown.

To exam the sensitivity of the model to the initial condition, we investigated how this impacts its predicting potential. Figure 3A shows predictions by the model for the second stand of the first site (Fabrikschleichach) when we add an error on the initial condition for *r*_0_ between −20% and +20% (in blue) with an increment of 4%. We also show the effect of combining this error on *r*_0_ with a −20% error on *h*_0_ (in cyan) and a +20% error on *h*_0_ (in magenta). Experimental measurements are represented in red. In Figure 3B we show the result of the model with a full range of error for *r*_0_ and *h*_0_ between −20% and +20% with an increment of 4%, while the experimental measurements are again represented in red. In these simulations some knowledge on the experimental measurement is still necessary as we need to known the allometric relationship *a priori*. However, we could avoid this assumption by estimating it for instance using the allometric relationship of the first stand used for partial fitting. Figure 3B would not change much: the result of this procedure is given in Figure 3C. Of course the prediction is likely to be more precise if we knew more than the single initial point. The existing but small dispersion of the model prediction due to variation in the initial condition as demonstrated by the panels in Figure 3, is encouraging. It appears that the sensitivity or our model to the initial condition is not a significant concern regarding the ability of the model to reproduce actual behavior.

Finally, mortality is not addressed by the model, although the model does produce cessation of growth under stressful circumstances. Therefore, tree mortality due to critical events (e.g., disturbance, pathogens, etc.) other than a limit on growth due to the external conditions, can create discontinuities in the data which are not captured by the model. Although the continual use of the 100 trees with largest DBHs tends to average this discontinuity (as the probability that a large change in those 100 trees occurs in 1 year is low), this might have created another limit to the accuracy. For instance, we note that we have observed in the 2010 measurements, i.e., after 188 years, the death of several of the 100 trees with the largest DBHs, which seems to have caused a decline of the average height of the 100 living trees with the largest DBHs as taller trees were replaced by smaller trees in this group, which is a result of size-related mortality dynamics (cf. Holzwarth et al., 2013).

If we wanted to address further this question, especially if we wanted to simulate many individual trees that interact together in a forest, we could multiply the height *h* by a random process *M*(*t*) that would be equal to 1 when the tree is alive and 0 when the tree is dead. Then the probability p(M(t) = 0|M(s<t) = 1) of transition between a living tree and a dead tree could be derived using experimental measurements and even be a function of the size of the tree. So far, though, this doesn't seem to be needed in the present application.

So far, the only things we assumed to be known for predicting the height are the external variables (i.e., temperature, water potential, PAR, and CO_2_ concentration), the species of the tree, and the allometric relationships for each stand. No other knowledge about the trees was used to perform the predictions. Measurements in themselves were used to compare our predictions with reality.

**Figure 4:**
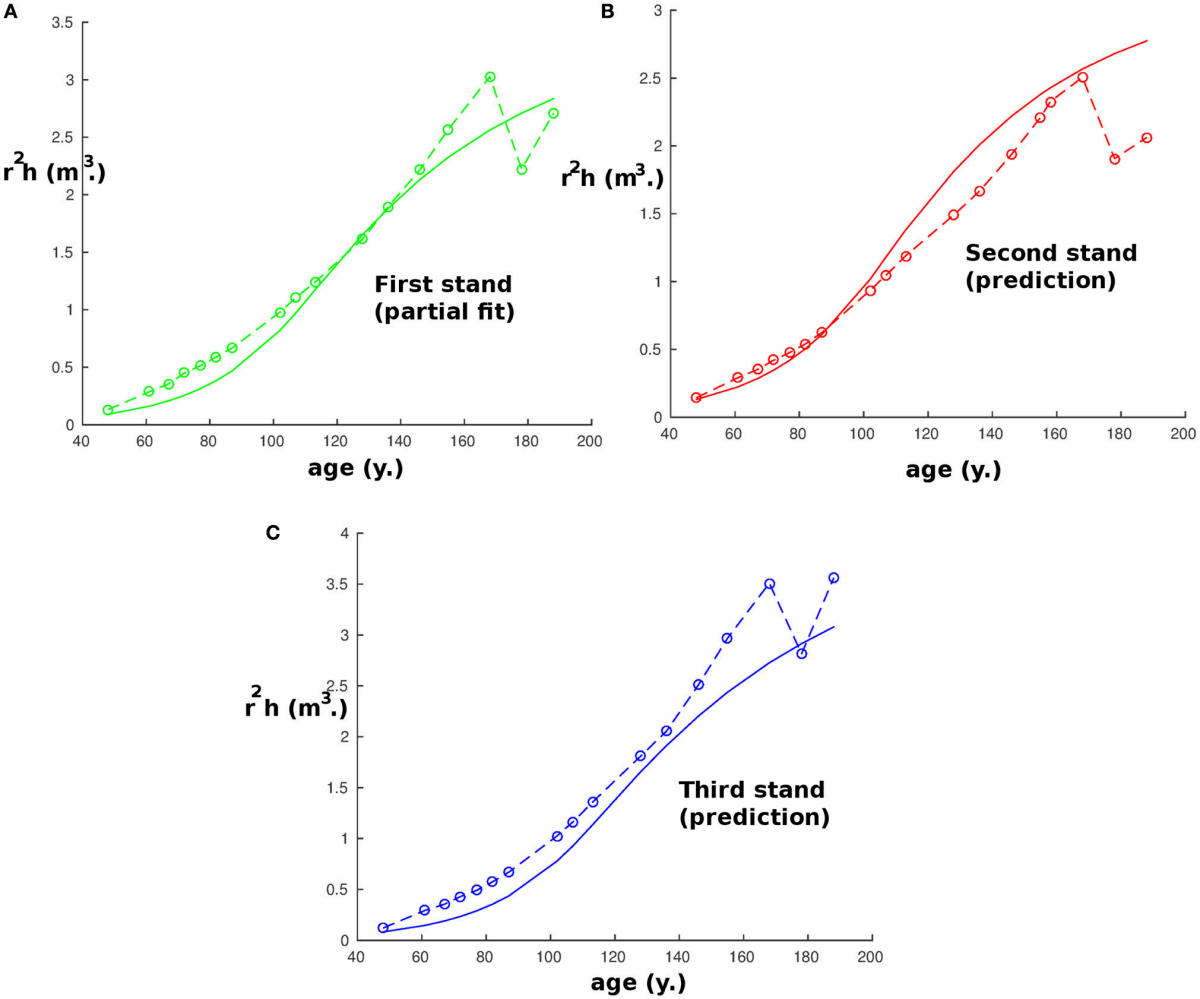
**Plot of the stem volume with age at the first site (Fabrikschleichach)**. Continuous curves are computed with the model and start when the tree density is lower than 1,000 tree ha^−1^, while circles correspond to experimental measurements. Panel **(A)** represents the partial fit on the reference stand. Panels **(B)** and **(C)** represent the results of the prediction with the parameters obtained by partial fitting.

We chose to predict height as there is usually a relatively small error of measurement in height compared to other variables. It should be noted that the same procedure is possible taking instead the stem volume as the quantity to predict. However, the results would be less precise because they will be susceptible to cumulative measurement errors of the radius and the stem height, among other reasons, but they would still be accurate (the highest error is around 25% after 75 years) and are presented in Figure 4. As in Figure 1, we find again between 168 and 188 years that the effect of thinning reduces the precision and explains the apparently strange experimental values. Also, we chose to predict height instead of radius or diameter as the measurements performed on the radius were the DBH which might be different from the equivalent cylindrical diameter, especially for the 100 trees with the largest DBH, and this would have added an artificial error of measurement.

### 3.1. Notable behavior

The results of this model are very encouraging and are not limited to predicting the height of dominant trees but show notable qualitative behaviors in other respects.

**Figure 5:**
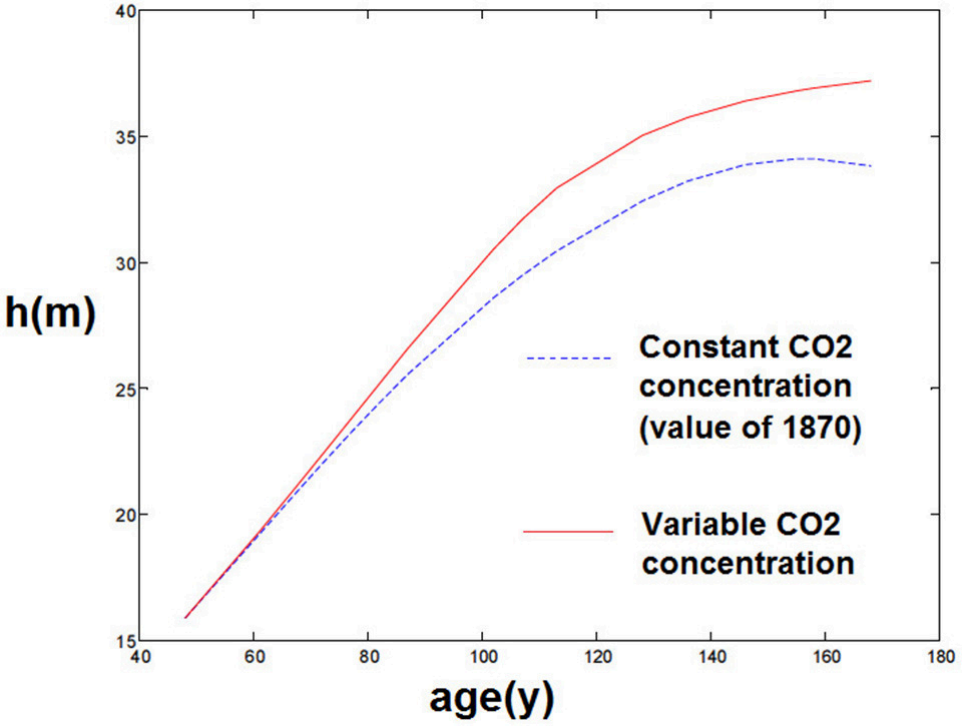
**Comparison of simulations with and without increasing atmospheric CO_2_**.

Firstly, it is known that forest stand growth dynamics have significantly changed since 1870, as shown in Pretzsch et al. (2014), and many experiments have been conducted to measure the impact of climate and CO_2_ change on forests. Climate and CO_2_ change also shows an impact on this individual tree model, as the model depends on external conditions such as temperature and soil moisture, as well as CO_2_ concentration. For instance, using the model with a constant CO_2_ concentration (from 1870) rather than the actual CO_2_ concentrations gives biased results and a slower growth of trees (Figure 5)^2^. This seems coherent with both the idea that atmospheric CO_2_ had an impact on forest stand growth and that present forest stands are growing more rapidly than comparable stands before.

Also, if we consider a small tree in a forest, shaded by the canopy above, it would receive only a small amount of light. Therefore, the model would predict a very limited maximum height and the tree would remain near this height with very small growth until a big tree nearby falls. Then the light received would be higher and enough for the tree to grow taller very quickly. If the tree were very close to the dead big tree then the aperture is large enough and the tree would reach the canopy. If not, and if the tree is still significantly partially shaded compared to the rest of the trees, then its limit height would be lower than the forest height and it would reduce its growth progressively until it were close to this height. This behavior would seem to be qualitatively in accordance with reality Nagel et al. (2007), but due to a lack of data we have not been able test this quantitatively.

**Figure 6:**
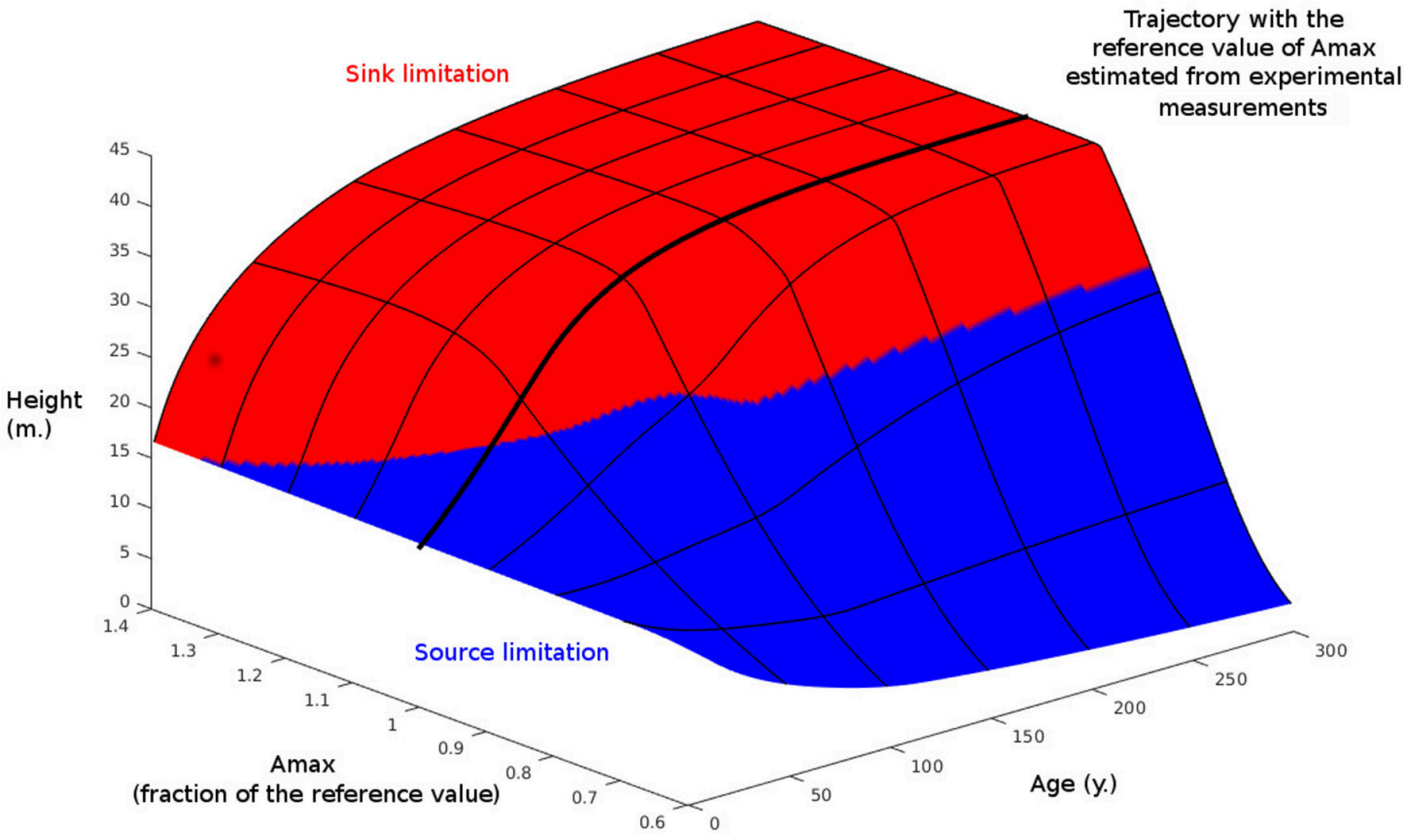
**Effect of maximal carbon assimilation (*A*_max_) and time on simulated tree height**. Also shown is the limiting process for growth: source or sink. *A*_max_ is normalized to 1 at its value in Table 1.

Finally, we have examined the response of our model to a wide range of maximal carbon assimilation parameter values (*A*_max_) in order to investigate the conditions under which sink-limited growth dominates (Figure 6). Under the reference simulation, the growth of the tree was source-limited when young, but switched to being sink-limited when it reached 84 y old (this simulation is equivalent to that presented in Figure 1B, with *A*_max_ obtained after a partial fit on the first stand from the value in Table 1. At low values of *A*_max_ (i.e., approximately <80% of the reference value), the tree remained source-limited to maturity, whereas at high values it was sink-limited for most of its life.

**Figure 7:**
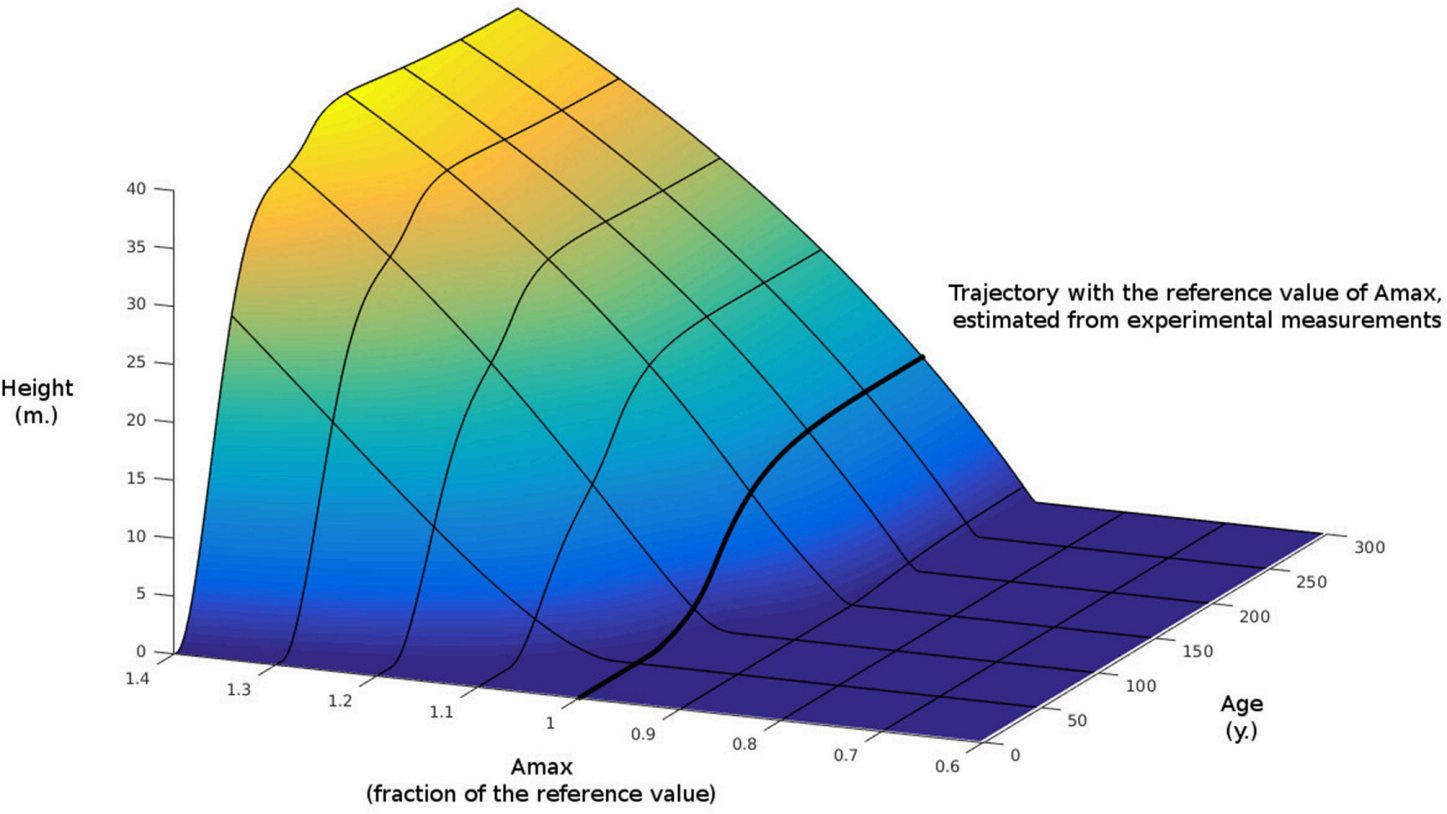
**Difference in height growth with Figure 6 when sink-limited growth is switched off**.

To examine further the idea of the importance of considering the sink limit in this model we performed a similar simulation but we switched off the sink limit, letting photosynthesis being the only limiting factor. We then computed the difference of the two simulations. This difference is shown in Figure 7. As we can see for regions where *A*_max_ is large, the difference in tree heights between the two simulations can reach around 40 m after several 100 years.

## 4. Discussion

Our aim in developing this model has been to provide a means of representing realistic tree growth and forest/vegetation dynamics at the global scale. To simulate individual tree competition our preferred method is to use a gap model of succession. Our global gap-model, HYBRID (Friend and White, 2000), computes individual-level photosynthesis and allocates photosynthate to leaves, stems, and roots, without consideration of sink limitations. All other DGVMs do something similar (Fatichi et al., 2014), usually at the big-leaf level (i.e., they do not consider individuals at all). The motivation of the new model described here is to explore a framework for introducing, in DGVMs, sink-limited growth. We have suggested as simple a scheme as possible to avoid reaching the point where we can no longer simulate dynamics at the global scale due to limited computing resources. The approach to modeling photosynthesis in DGVMs is similar to ours, in that the whole canopy is typically simulated as one leaf, and so it seems appropriate to simulate the meristems with a similar approach. We have chosen two meristem types—apical and lateral, in order to simulate the essential properties of height and diameter growth. Our new model is a major advance over current global models, and we expect that it could have significant consequences for our understanding of the historical and future global terrestrial carbon sink.

Our model starts from physiological considerations about trees and several hypotheses to derive differential equations that are solved over time to obtain the growth of a tree and predict its size. Although the model is fairly simple and far from taking every possible parameter of the tree into account, it seems to obtain very good results and predictions which agree closely with observations from different stands of trees with different environmental conditions.

Interestingly, our approach gives rise to two limiting tree heights: *h*_1_, the limit height above which photosynthesis cannot occur, and *h*_2_, the height above which growth is limited by the capacity of the meristematic regions themselves to grow at low water potential. These two heights represent different processes: *h*_1_ includes the factors that limit photosynthesis such as minimal turgor potential and possible xylem cavitation (i.e., source limitations), whereas *h*_2_ takes into account intrinsic meristem factors such as the physiological limits of cell division and growth limits such as cell wall extension when the water potential is too low (i.e., sink limitations).

While *h*_1_ (and, more generally, source processes) is often considered as the only limiting height to explain the maximal height of trees (e.g., Koch et al., 2004), under some external conditions of light and temperature it could be that *h*_2_ (i.e., sink activity) becomes limiting (cf. Friend, 1993). Although in this model, water potential is still the critical factor which limits the height of trees, the amount of light received and the temperature during the growth period also play roles that could produce one or other limit and influence its value. Hence, our approach could give a new and more complete understanding of the limits of tree height, and growth more generally, through a balanced consideration of both source and sink limitations.

Concerning the derivation of the results, we could have avoided assuming known allometric relationships for each stand and tried to estimate them as described in Section 2.3 (“Control mechanism of the tree"). Another way to estimate the allometric relationship on the second and third stand could be to estimate it close to the allometric relathionship of the first stand used for partial fitting. In that case we would still get good results with *R*^2^ around 0.8 − 0.9. The small difference of accuracy between this case and the previous one is mainly due to the model becoming more sensitive to measurement error when we estimate the allometric relationship, and also to the estimated allometric relationship being a constant as explained in Section 2.3, whereas the allometric relationship in reality changes with changes in tree density and therefore time. Lastly, our model for α_2_ is probably too simple as we also explained in Section 2.3. Nevertheless, we can note that the accuracy of the results is still very good considering that we try here to predict the heights of three stands without knowing anything but the species, the CO_2_ concentration, the temperature, the soil moisture, the PAR, the number of trees per ha, and the initial tree dimensions. This last point also underscores an additional motivation to build such a relatively simple model: collecting data about the external variables can be difficult. Therefore, data can be limiting and in order to be practical the model should stay as simple as it can relative to the required data, provided that it can still give accurate results.

As a first dynamic model for an individual tree using such considerations about control mechanism and the maximal meristem-sustained limited rate of growth, improvements could probably be made by introducing additional parameters, especially taking the roots into account explicitly, and improving the function of the red:far-red ratio. Nevertheless, the model already gives very good results while being relatively simple, allowing its wider use such as through allowing many trees to interact with each other via shading, water potential, etc., and obtaining a model for the whole forest. So far the partial fitting of the parameters within bounds of 20% takes less than 120 s on a desk-top computer, and computing height over 100 y less than 0.1 s, which suggests that its use as a growth simulator within a forest model could be achieved with today's computer resources.

Our model makes the interesting prediction that under standard conditions/parameterization, trees shift from being source- to sink-limited as they mature. This behavior is consistent with field and experimental evidence for a lack of stem growth response to elevated CO_2_ in mature trees, despite increased photosynthesis (e.g., Körner, 2005; van der Sleen et al., 2014), whereas young fast growing trees show significant responses (e.g., Norby et al., 1999). In our model, the cause of the change in growth limitation as trees grow appears to be a combination of the effect of the limiting height for meristem activity, *h*_2_, and the dependency of the balance of photosynthesis and respiration on height.

The relative simplicity of the model and its extremely encouraging results should stimulate further use of similar differential models in the future. Starting from physiological considerations to derive differential equations and an individual tree model as a cornerstone for simulating a whole forest may considerably improve our understanding of forest behavior, including, but not limited to, the prediction of overall responses of forests to increasing CO_2_ concentrations in order to address the future terrestrial carbon sink.

A number of other models have the potential to address the issues considered in this paper. For example, the L-PEACH FSPM (Allen et al., 2005) contains a number of features that make it applicable to the source-sink debate, including carbohydrate storage dynamics, product inhibition of photosynthesis, a transport-resistance framework for sugar partitioning, a sink growth control from available carbon, a direct effect of water supply, and an intrinsic size limit. The pipe model constraint is used, making overall growth a strong function of leaf growth, which itself is determined by carbon supply. However, it is not clear the extent to which this model would predict either source- or sink-limitations under different environmental conditions and/or timescales. This would be a very interesting exercise. FSPMs are also relevant to the calculation of canopy red:far red ratios as they explicitly treat light interception throughout the canopy. In fact, some herbaceous crop models already compute the canopy red:far red ratio distribution and its influence on morphogenesis (e.g., Chelle et al., 2007). Such approaches could be simplified for implementing dynamic red:far red ratio responses in our model.

To conclude, we have proposed a differential model for modeling tree growth over time under external conditions such as temperature, soil moisture, and CO_2_ concentration. This model takes into account not only photosynthesis and the carbon balance but also meristem behavior and cellular growth limits. We established a procedure to parametrize the model with measurable quantities and reference measurements. This model seems not only to fit data very well but also to give accurate predictions for tree height and tree volume.

## Author contributions

AH executed the study designed by AF with input from AH-P and TR. HP provided data for model evaluation. AH initially wrote the manuscript and all authors commented on and edited the manuscript.

## Funding

AH wishes to thank the Cambridge Faculty of Mathematics for support via the PMP scheme.

### Conflict of interest statement

The authors declare that the research was conducted in the absence of any commercial or financial relationships that could be construed as a potential conflict of interest.

## Footnotes

^1^Variation of α_2_ could strongly increase the complexity of the equations as α is an exponent in our model.

^2^The three last experimental points are likely to be decreasing because of the thinning.
